# Clinical predictors of NEDA-3 one year after diagnosis of pediatric multiple sclerosis: an exploratory single-center study

**DOI:** 10.3389/fnins.2023.1259306

**Published:** 2023-09-14

**Authors:** Filipe Palavra, Diogo Silva, Catarina Fernandes, Ricardo Faustino, Mónica Vasconcelos, Cristina Pereira, Carmen Costa, Joana Afonso Ribeiro, Joana Amaral, Conceição Robalo

**Affiliations:** ^1^Center for Child Development–Neuropediatrics Unit, Hospital Pediátrico, Centro Hospitalar e Universitário de Coimbra, Coimbra, Portugal; ^2^Laboratory of Pharmacology and Experimental Therapeutics, Faculty of Medicine, Coimbra Institute for Clinical and Biomedical Research (iCBR), University of Coimbra, Coimbra, Portugal; ^3^Clinical Academic Center of Coimbra, Coimbra, Portugal; ^4^Faculty of Medicine, University of Coimbra, Coimbra, Portugal; ^5^Neurology Department, Centro Hospitalar e Universitário de Coimbra, Coimbra, Portugal; ^6^Ribeiro Sanches Higher School of Health, Research Group in Health Sciences and Technologies–NICiTeS, Polytechnic Institute of Lusophony (IPLuso), Lisboa, Portugal; ^7^Portuguese Red Cross Higher Health School (ESSCVP), Lisboa, Portugal; ^8^Biomedical Research Group (BioRG), Faculty of Engineering and Faculty of Veterinary Medicine, Lusófona University, Lisboa, Portugal

**Keywords:** multiple sclerosis, children, adolescents, NEDA-3, predictors

## Abstract

**Introduction:**

Multiple sclerosis (MS) is an inflammatory and demyelinating disorder of central nervous system that can be diagnosed in pediatric age (<18 years) in 3–5% of the cases. This early onset is associated with higher relapse rates and earlier progression to neurological disability. By using NEDA-3 (No Evidence of Disease Activity-3) criteria, we aimed to identify clinical predictors associated with absence of disease activity and control of disease progression 12 months after the diagnosis, in a cohort of pediatric-onset MS (POMS) patients regularly followed-up in our center.

**Methods:**

We analyzed demographic, clinical, laboratorial and imaging variables of patients with POMS identified in our center, between 2010 and 2021, in two moments: at the diagnosis and 12 months after it. Statistical tests were applied to compare the distribution of those variables between groups defined by NEDA-3 status and by each one of its three variable components.

**Results:**

We included 27 patients in the study (18 female), with a mean age of 14.8 years (± 2.8), being all diagnosed with relapsing–remitting MS and with a median score of 1.5 at the Expanded Disability Status Scale (EDSS). The use of natalizumab (*p* = 0.017) and the negativity for anti-EBV IgG antibodies (*p* = 0.018) at diagnosis were associated with a higher achievement of NEDA-3 status 12 months after, in our cohort. Prescribed treatment was also associated with statistically significant differences in the “absence of MRI activity” component of NEDA-3 (*p* = 0.006): patients under treatment with natalizumab had a higher probability of achieving this status, and the opposite was observed in glatiramer acetate-treated children.

**Discussion and conclusion:**

Our exploratory results underline the pivotal importance that an early and more effective therapeutical approach may have in the control of disease activity, in POMS. Additionally, they also seem to suggest that the presence of anti-EBV antibodies is not innocent, as it can be related to a less favorable evolution of the disease, even at a very early stage. Further studies are needed to confirm the applicability of these variables as prognostic and personalized tools in this clinical setting.

## Introduction

Multiple sclerosis (MS) is a chronic, inflammatory and primary demyelinating disorder of central nervous system (CNS), whose most frequent presentation is based on the occurrence of clinical relapses, that can, as they happen, condition the accumulation of neurological disability (Renoux et al., 2007; Compston and Coles, 2008). In about 3–5% of cases, the onset occurs in pediatric age (i.e., before 18 years old; Mrosková et al., 2021) and is associated with some particular features, different from what is normally seen in adults with the disease. In pediatric-onset MS (POMS), there is an earlier progression to irreversible neurological disability (despite a longer interval of progression), a more significant imaging expression of the disease and a higher annualized relapse rate, at least in the first few years after diagnosis (Brenton et al., 2020; Fisher et al., 2020; De Meo et al., 2021). In fact, the relapsing–remitting form of MS is the most frequently diagnosed in children and adolescents, occurring in nearly 98% of cases (Fisher et al., 2020).

POMS diagnosis follows the same precepts established for adults, being based on McDonald criteria (the 2017 review is currently in practical evidence; Thompson et al., 2018) and on the recommendations of the International Pediatric MS Study Group (IPMSSG; Krupp et al., 2013).

Established in 2009 as a true target of the therapeutic intervention strategy (Havrdova et al., 2009), the concept of NEDA (No Evidence of Disease Activity) has evolved over time and has been adding variables that, more recently, focus on 3 fundamental aspects (NEDA-3): absence of clinical relapses, absence of progression in neurological disability, as measured by the Expanded Disability Status Scale (EDSS; Kurtzke, 1983), and absence of imaging activity of the disease, using Magnetic Resonance Imaging (MRI). This metric has been used as a prognostic indicator and as a way of assessing the effectiveness of immunomodulatory therapy in the disease, having become an interesting work tool in clinical practice, at least in the adult population (Diem et al., 2018; Prosperini et al., 2018; De Carvalho et al., 2021; Guerra et al., 2021).

Therapeutic trials are not as abundant in POMS, which is why there are not many studies that evaluate the performance of NEDA-3 itself in the pediatric population (Yeshokumar et al., 2017). In this study, by using NEDA-3 criteria, we aimed to identify clinical predictors associated with absence of disease activity and control of disease progression 12 months after the diagnosis, in a cohort of POMS patients regularly followed-up in our center.

## Methods

### Study design and participants

To perform this observational, retrospective, exploratory and unicentric study, we identified and selected patients with the diagnosis of POMS, followed in our center (Hospital Pediátrico, Centro Hospitalar e Universitário de Coimbra, Portugal), from 2010 to 2021. We considered the following inclusion criteria: (1) children or adolescents under the age of 18, with a confirmed diagnosis of MS (established according to McDonald 2010/2017 criteria) for at least 1 year and who underwent MRI at diagnosis and 12 months later; (2) signature of the Informed Consent form. This study was approved by the local Ethics Committee, complying with all the requirements of good clinical practice.

### Data collection

We collected the following data from the clinical records available:

- Biodemographic variables: age at diagnosis, gender, family history of MS, Body Mass Index (BMI) at diagnosis.
- Clinical, laboratorial and imaging variables at the moment of MS diagnosis: clinical form of disease, number of relapses occurred previously to diagnosis, topography of relapses occurred previously to diagnosis, EDSS score, established disease-modifying therapy, concomitant chronic diseases, evidence of oligoclonal bands in cerebrospinal fluid (CSF) analysis (isoelectric focusing), evidence of anti-EBV (Epstein–Barr virus) IgG antibodies, evidence of anti-CMV (Cytomegalovirus) IgG antibodies, serum levels of vitamin D, number of lesions in T2-weighted images on MRI, presence and number of gadolinium-enhanced lesions on MRI and presence of tumefactive lesions on MRI.
- Clinical and laboratorial variables at 12 months after MS diagnosis, in order to evaluate NEDA-3 status: number of clinical relapses occurred during the period of 12 months post-diagnosis, EDSS score at the end of that time period, MRI activity of disease (through the presence/number of new or enlarged lesions in T2-weighted images, presence/number of gadolinium-enhanced lesions and presence of tumefactive lesions) and achievement of NEDA-3 status.

The variable “number of lesions in T2-weighted images on MRI at the moment of MS diagnosis” was categorized as “1–4,” “5–9” and “≥ 10,” the variable “number of gadolinium-enhanced lesions on MRI at the moment of MS diagnosis” as “1–2,” “3–4” and “≥ 5” and the variable “number of new or enlarged lesions in T2-weighted images on MRI, at 12 months after MS diagnosis” as “0,” “1–4,” “5–9” and “≥ 10.” The EDSS score was used to evaluate the level of neurological disability. NEDA-3 status was defined through the simultaneous presence of the following criteria: (1) absence of clinical relapses (absence of new neurological signs or symptoms persisting for, at least, 24 h, excluding a concomitant disease); (2) absence of progression in neurological disability (absence of an EDSS score increase in ≥1 point from baseline EDSS, at diagnosis); and (3) absence of MRI activity (absence of new or enlarged lesions in T2-weighted images or gadolinium-enhanced lesions). Patients lacking data for the assessment of at least 1 of these 3 criteria were considered as not having achieved NEDA-3.

### Statistical analysis

Patients were divided into two groups: (1) those who achieved NEDA-3 status at 12 months after POMS diagnosis (NEDA-3 +); and (2) those who did not achieve it, in the same period of time (NEDA-3 -). Both groups were analyzed and compared regarding the influence of the collected variables on NEDA-3 achievement. We also tested for the existence of a statistically significant association between all the variables at diagnosis and the attainment of each one of the 3 necessary criteria for the definition of NEDA-3. Statistical analysis was performed using the Statistical Package for the Social Sciences software (SPSS®), version 26.0. In the descriptive analysis, continuous variables were presented as mean and standard deviation or median, and categorical variables in the form of relative and absolute frequency. Continuous variables were compared within and between groups, using independent t-test or Mann-Whittney U test, depending on the results of previous normality tests. Categorical variables were compared within and between groups, using Chi-squared test (*X*^2^) or Fisher exact test. A *post-hoc* analysis was also conducted, to evaluate the distribution of adjusted standardized residuals, to identify the sub-variables with statistical significance. We considered statistically significant a value of *p* < 0.05.

## Results

Our study included 27 patients with POMS. Table 1 shows the distribution of the studied demographic, clinical, laboratorial and imaging variables, at the moment of MS diagnosis.

**Table 1:** Descriptive analysis of demographic, clinical, laboratorial and imaging data at POMS diagnosis.

Variable			
Mean age (years)			14.8 ± 2.8 *(n = 27)*
Gender	Female		66.7% *(n = 18)*
	Male		33.3% *(n = 9)*
Family history of multiple sclerosis *(n = 26) ^(1)^*	Yes		15.4% *(n = 4)*
	No		84.6% *(n = 22)*
Clinical form	RRMS		100% *(n = 27)*
Mean Body Mass Index (kg/m^2^)			20.7 ± 3.6 *(n = 13) ^(1)^*
Mean number of relapses occurred previously to diagnosis			1.3 ± 0.5 *(n = 27)*
Topography of relapses occurred previously to diagnosis	Optic nerve		44.4% *(n = 12)*
	Spinal cord		33.3% *(n = 9)*
	Brainstem		7.4% *(n = 2)*
	Hemispherical		3.7% *(n = 1)*
	Optic nerve and brainstem		11.1% *(n = 3)*
Median EDSS score			1.5 *(n = 27)*
Prescribed DMT	None		18.5% *(n = 5)*
	Interferon beta-1a		29.6% *(n = 8)*
	Interferon beta-1b		7.4% *(n = 2)*
	Pegylated interferon beta-1a		3.7% *(n = 1)*
	Natalizumab		14.8% *(n = 4)*
	Glatiramer acetate		18.5% *(n = 5)*
	Teriflunomide		3.7% *(n = 1)*
	Fingolimod		3.7% *(n = 1)*
Concomitant chronic diseases	Absent		74.1% *(n = 20)*
	Present 25.9% *(n = 7)*	Asthma	18.5% *(n = 5)*
		Allergic rhinitis	7.4% *(n = 2)*
		Atopy	7.4% *(n = 2)*
		Chronic sinusitis	3.7% *(n = 1)*
Mean serum levels of vitamin D (mg/dL)			23.6 ± 9.7 *(n = 11) ^(1)^*
Oligoclonal bands in CSF *(n = 22) ^(1)^*	Present		72.7% *(n = 16)*
	Absent		27.3% *(n = 6)*
Anti-EBV IgG antibodies *(n = 11) ^(1)^*	Present		81.8% *(n = 9)*
	Absent		18.2% *(n = 2)*
Anti-CMV IgG antibodies *(n = 11) ^(1)^*	Present		45.5% *(n = 5)*
	Absent		54.5% *(n = 6)*
Number of lesions in T2-weighted images on MRI *(n = 26) ^(1)^*	1–4		11.5% *(n = 3)*
	5–9		23.1% *(n = 6)*
	≥ 10		65.4% *(n = 17)*
Presence and number of gadolinium-enhanced lesions on MRI *(n = 26) ^(1)^*	No		30.8% *(n = 8)*
	Yes 69.2% *(n = 18)*	1–2	38.5% *(n = 10)*
		3–4	15.4% *(n = 4)*
		≥ 5	15.4% *(n = 4)*
Tumefactive lesions on MRI *(n = 26) ^(1)^*	Present		3.8% *(n = 1)*
	Absent		96.2% *(n = 25)*

(1) Reduced n value due to missing data. CSF, cerebrospinal fluid; CMV, cytomegalovirus; DMT, disease-modifying therapy; EBV, Epstein–Barr virus; EDSS, Expanded Disability Status Scale; MRI, magnetic resonance imaging; RRMS, relapsing–remitting multiple sclerosis.

The mean age of the patients was 14.8 (± 2.8) years, most of them were females (*n* = 18; 66.7%) and did not have family history of MS (*n* = 22; 84.6%). All patients presented with a relapsing–remitting form of the disease. The mean number of relapses before diagnosis was 1.3 (± 0.5), being 44.4% (*n* = 12) of them solely localized at the optic nerve (optic neuritis). The patients had a median EDSS score of 1.5 points and 74.1% (*n* = 20) did not suffer from a concomitant chronic disease. Sixty three percent (*n* = 17) of the participants started immunomodulatory therapy (interferon beta, glatiramer acetate or teriflunomide) and 18.5% (*n* = 5) began more potent drugs, considered second-line (natalizumab or fingolimod). In 18.5% (*n* = 5) of the cases, it was decided not to start any treatment (essentially by decision of the family, when children under 10 years of age were involved). Most of the patients had 10 or more lesions in T2-weighted images on MRI (*n* = 17; 65.4%) and in 69.2% (*n* = 18) gadolinium-enhanced lesions were identified in the first scan. Only 1 patient presented with tumefactive lesions.

**Figure 1:**
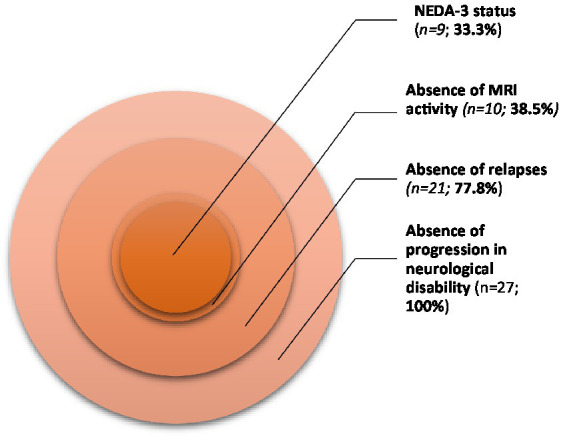
Distribution of NEDA-3 status and its defining criteria in our population.

Table 2; Figure 1 represent the distribution of the studied clinical and imaging variables at 12 months after the diagnosis of POMS, as well as the attainment of NEDA-3 status and respective defining criteria at the same time point.

**Table 2:** Descriptive analysis of clinical and imaging data and attainment of NEDA-3 status (and respective defining criteria), at 12 months after POMS diagnosis.

Variable			
Absence of clinical relapses during the first 12 months after diagnosis	Criterion was met	77.8% *(n = 21)*	
	Criterion was not met	22.2% *(n = 6)*	
	Mean number of clinical relapses since diagnosis	0.4 ± 0.8	
Absence of progression in neurological disability (EDSS) during the first 12 months after diagnosis	Criterion was met	100.0% *(n = 27)*	
	Criterion was not met	0.0% *(n = 0)*	
	Median EDSS score (points)	1.5	
Absence of MRI activity of disease during the first 12 months after diagnosis *(n = 26) ^(1)^*	Criterion was met	38.5% *(n = 10)*	
	Criterion was not met	61.5% *(n = 16)*	
	New or enlarged lesions in T2-weighted images on MRI *(n = 25) ^(1)^*	0	44.0% *(n = 11)*
		1–4	20.0% *(n = 5)*
		5–9	24.0% *(n = 6)*
		≥ 10	12.0% *(n = 3)*
	Gadolinium-enhanced lesions on MRI *(n = 25) ^(1)^*	Present	28.0% *(n = 7)*
		Absent	72.0% *(n = 18)*
	Mean number of gadolinium-enhanced lesions on MRI *(n = 25) ^(1)^*	0.5 ± 0.9	
	Tumefactive lesions on MRI *(n = 25) ^(1)^*	Present	0% *(n = 0)*
		Absent	100% *(n = 25)*
NEDA-3 status ^(2)^ *(n = 27)*	Met	33.3% *(n = 9)*	
	Not met	66.7% *(n = 18)*	

(1) Reduced n value due to missing data. (2) Patients lacking data about any of NEDA-3 criteria were classified as not achieving this status. EDSS, Expanded Disability Status Scale; MRI, magnetic resonance imaging; NEDA-3, No Evidence of Disease Activity-3.

One third of the patients (33.3%; *n* = 9) attained NEDA-3 status at 12 months after MS diagnosis. Regarding the 3 needed criteria to verify this condition, 77.8% (*n* = 21) of the patients met the “absence of clinical relapses” criterion and all of them (*n* = 27) met the “absence of progression in neurological disability” criterion, according to EDSS scores. However, most participants (61.5%; *n* = 16) did not meet conditions to verify the “absence of MRI activity of disease” criterion.

Table 3 compares the previously mentioned demographic, clinical, laboratorial and imaging variables between the groups defined by NEDA-3 status (NEDA-3 + and NEDA-3 -), evaluated at 12 months after POMS diagnosis. The variable “clinical form of disease” was not compared, as it was the same in all patients.

**Table 3:** Comparison of variables between groups defined by NEDA-3 status.

Variable			NEDA-3 + ^(2)^	NEDA-3 – ^(2)^	*p*
Age at diagnosis (years)			15.0	16.0	NS (0.253)
Gender	Female		33.3% *(n = 6)*	66.7% *(n = 12)*	NS (1.000)
	Male		33.3% *(n = 3)*	66.7% *(n = 6)*	
Family history of MS *(n = 26) ^(1)^*	Yes		25.0% *(n = 1)*	75.0% *(n = 3)*	NS (1.000)
	No		36.4% *(n = 8)*	63.6% *(n = 14)*	
BMI at diagnosis (kg/m^2^)			20.3 ± 3,6	20.9 ± 3,9	NS (0.769)
Number of clinical relapses occurred previously to diagnosis			1.0	1.0	NS (0.980)
Topography of clinical relapses occurred previously to diagnosis	Optic nerve		16.7% *(n = 2)*	83.3% *(n = 10)*	NS (0.287)
	Spinal cord		44.4% *(n = 4)*	55.6% *(n = 5)*	
	Brainstem		50.0% *(n = 1)*	50.0% *(n = 1)*	
	Hemispherical		0.0% *(n = 0)*	100.0% *(n = 1)*	
	Optic nerve and brainstem		66.7% *(n = 2)*	33.3% *(n = 1)*	
EDSS score			1.5	1.5	NS (1.000)
Established disease-modifying therapy	None		60.0% *(n = 3)*	40.0% *(n = 2)*	**0.017** ***X***^**2**^ **= 13.032** **φ**_**C**_ **= 0.742**
	Interferon beta-1a		25.0% *(n = 2)*	75.0% *(n = 6)*	
	Interferon beta-1b		0.0% *(n = 0)*	100.0% *(n = 2)*	
	Pegylated interferon beta-1a		0.0% *(n = 0)*	100.0% *(n = 1)*	
	Natalizumab		100.0% *(n = 4)*	0.0% *(n = 0)*	
	Glatiramer acetate		0.0% *(n = 0)*	100.0% *(n = 5)*	
	Teriflunomide		0.0% *(n = 0)*	100.0% *(n = 1)*	
	Fingolimod		0.0% *(n = 0)*	100.0% *(n = 1)*	
Chronic diseases	Absent		45.0% *(n = 9)*	55.0% *(n = 11)*	NS (0.059)
	Present		0.0% *(n = 0)*	100.0% *(n = 7)*	
Serum levels of vitamin D (mg/dL)			20.4 ± 6.1	26.2 ± 11.9	NS (0.355)
Oligoclonal bands in CSF *(n = 22) ^(1)^*	Present		37.5% *(n = 6)*	62.5% *(n = 10)*	NS (0.655)
	Absent		50.0% *(n = 3)*	50.0% *(n = 3)*	
Anti-EBV IgG antibodies *(n = 11) ^(1)^*	Present		0.0% *(n = 0)*	100.0% *(n = 9)*	**0.018** ***Χ***^**2**^ **= 11.000** **φ = 1.000**
	Absent		100.0% *(n = 2)*	0.0% *(n = 0)*	
Anti-CMV IgG antibodies *(n = 11) ^(1)^*	Present		20.0% *(n = 1)*	80.0% *(n = 4)*	NS (1.000)
	Absent		33.3% *(n = 2)*	66.6% *(n = 4)*	
Number of lesions in T2-weighted images on MRI at diagnosis *(n = 26) ^(1)^*	0–4		66.7% *(n = 2)*	33.3% *(n = 1)*	NS (0.498)
	5–9		33.3% *(n = 2)*	66.7% *(n = 4)*	
	≥ 10		29.4% *(n = 5)*	70.6% *(n = 12)*	
Presence and number of gadolinium-enhanced lesions on MRI at diagnosis *(n = 26) ^(1)^*	No		37.5% *(n = 3)*	62.5% *(n = 5)*	NS (0.940)
	Yes	1–2	30.0% *(n = 3)*	70.0% *(n = 7)*	
		3–4	25.0% *(n = 1)*	75.0% *(n = 3)*	
		≥ 5	50.0% *(n = 2)*	50.0% *(n = 2)*	
Tumefactive lesions on MRI at diagnosis *(n = 26) ^(1)^*	Present		100.0% *(n = 1)*	0.0% *(n = 0)*	NS (0.346)
	Absent		32.0% *(n = 8)*	68.0% *(n = 17)*	

(1) Reduced n value due to missing data. (2) Continuous variables are shown as mean + standard deviation (BMI at diagnosis and serum levels of vitamin D) or median. Categorical variables are shown as relative and absolute frequency. BMI, body mass index; CMV, Cytomegalovirus; CSF, cerebrospinal fluid; EBV, Epstein–Barr virus; EDSS, Expanded Disability Status Scale; MRI, magnetic resonance imaging; NS, not significant.Bold values are statistically significant.

Statistically significant differences between groups were observed regarding the disease-modifying therapy started at diagnosis (*X*^2^ = 13.032; *p* = 0.017). We verified that all patients under treatment with natalizumab (*n* = 4) achieved NEDA-3. This is supported by the existence of a statistically significant strong association (φ_C_ = 0.742) between the use of this monoclonal antibody and NEDA-3 achievement (with a module of the distribution of adjusted standardized residuals for natalizumab higher than 1.96 standard-deviations, given *α* = 0.05).

In parallel, we also found a statistically significant difference between groups regarding the presence of anti-EBV IgG antibodies (*X*^2^ = 11.000; *p* = 0.018), since all patients with confirmed negativity for these antibodies attained NEDA-3. The association between both variables was perfect (φ = 1.000).

No differences were found between groups considering the following variables: age at diagnosis, gender, family history of MS, BMI at diagnosis, number and topography of clinical relapses occurred previously to diagnosis, EDSS score at diagnosis, existence of chronic diseases, oligoclonal bands in CSF, anti-CMV IgG antibodies, number of lesions in T2-weighted images, gadolinium-enhanced lesions or tumefactive lesions on MRI (at diagnosis).

To potentiate the statistical power of our study, we evaluated the existence of associations between the variables measured at diagnosis and the individual achievement of each one of the three NEDA-3 criteria. Table 4 compares the variables collected at diagnosis between groups defined by the “absence of clinical relapses” and “absence of MRI activity of disease” criteria (both measured at 12 months after POMS diagnosis). We did not compare groups in function of the “absence of progression in neurological disability” criterion, since this variable had a constant distribution throughout our sample.

**Table 4:** Comparison of variables between patients clustered by the “absence of clinical relapses” and “absence of MRI activity of disease” criteria, at 12 months after MS diagnosis.

Variable			Patients without clinical relapses ^(2)^	Patients with clinical relapses ^(2)^	*p (clinical relapses)*	Patients without MRI activity of disease ^(2)^	Patients with MRI activity of disease ^(2)^	*p (MRI activity of disease)*
Age at diagnosis (years)			16.0	16.0	NS (0.670)	15.0	16.0	NS (0.391)
Gender *(n = 27/26) ^(1)^*	Female		72.2% *(n = 13)*	27.8% *(n = 5)*	NS (0.628)	41.2% *(n = 7)*	58.8% *(n = 10)*	NS (1.000)
	Male		88.9% *(n = 8)*	11.1% *(n = 1)*		33.3% *(n = 3)*	66.7% *(n = 6)*	
Family history of MS *(n = 26/25) ^(1)^*	Yes		75.0% *(n = 3)*	25.0% *(n = 1)*	NS (1.000)	25.0% *(n = 1)*	75.0% *(n = 3)*	NS (0.626)
	No		77.3% *(n = 17)*	22.7% *(n = 5)*		42.9% *(n = 9)*	57.1% *(n = 12)*	
BMI at diagnosis (kg/m^2^)			20.3 ± 3.6	21.5 ± 4.2	NS (0.614)	20.3 ± 3.6	20.2 ± 3.5	NS (0.965)
Number of clinical relapses occurred previously to diagnosis			1.00	1.00	NS (0.798)	1.00	1.00	NS (0.816)
Topography of clinical relapses occurred previously to diagnosis *(n = 27/26) ^(1)^*	Optic nerve		75.0% *(n = 9)*	25.0% *(n = 3)*	NS (1.000)	25.0% *(n = 3)*	75.0% *(n = 9)*	NS (0.260)
	Spinal cord		77.8% *(n = 7)*	22.2% *(n = 2)*		44.4% *(n = 4)*	55.6% *(n = 5)*	
	Brainstem		100.0% *(n = 2)*	0.0% *(n = 0)*		50.0% *(n = 1)*	50.0% *(n = 1)*	
	Hemispherical		100.0% *(n = 1)*	0.0% *(n = 0)*		0.0% *(n = 0)*	100.0% *(n = 1)*	
	Optic nerve and brainstem		66.7% *(n = 2)*	33,3% *(n = 1)*		100.0% *(n = 2)*	0.0% *(n = 0)*	
EDSS score			1.5	2.0	NS (0.408)	1.5	1.5	NS (0.816)
Established disease-modifying therapy *(n = 27/26) ^(1)^*	None		80.0% *(n = 4)*	20.0% *(n = 1)*	NS (0.372)	80.0% *(n = 4)*	20.0% *(n = 1)*	**0.006** ***X***^**2**^ **= 14.899** **φ**_**C**_ **= 0.799**
	Interferon beta-1a		75.0% *(n = 6)*	25.0% *(n = 2)*		28.6% *(n = 2)*	71.4% *(n = 5)*	
	Interferon beta-1b		100.0% *(n = 2)*	0.0% *(n = 0)*		0.0% *(n = 0)*	100.0% *(n = 2)*	
	Pegylated interferon beta-1a		0.0% *(n = 0)*	100.0% *(n = 1)*		0.0% *(n = 0)*	100.0% *(n = 1)*	
	Natalizumab		100.0% *(n = 4)*	0.0% *(n = 0)*		100.0% *(n = 4)*	0.0% *(n = 0)*	
	Glatiramer acetate		80.0% *(n = 4)*	20.0% *(n = 1)*		0.0% *(n = 0)*	100.0% *(n = 5)*	
	Teriflunomide		100.0% *(n = 1)*	0.0% *(n = 0)*		0.0% *(n = 0)*	100.0% *(n = 1)*	
	Fingolimod		0.0% *(n = 0)*	100.0% *(n = 1)*		0.0% *(n = 0)*	100.0% *(n = 1)*	
Chronic diseases *(n = 27/26) ^(1)^*	Absent		85.0% *(n = 17)*	15.0% *(n = 3)*	NS (0.290)	47.4% *(n = 9)*	52.6% *(n = 10)*	NS (0.190)
	Present		57.1% *(n = 4)*	42.9% *(n = 3)*		14.3% *(n = 1)*	85.7% *(n = 6)*	
Serum levels of vitamin D (mg/dL)			19.0	37.5	NS (0.145)	20.4 ± 6,1	26.0 ± 13.3	NS (0.417)
Oligoclonal bands in CSF *(n = 22/21) ^(1)^*	Present		75.0% *(n = 12)*	25.0% *(n = 4)*	NS (1.000)	43.8% *(n = 7)*	56.3% *(n = 9)*	NS (0.635)
	Absent		66.7% *(n = 4)*	33.3% *(n = 2)*		60.0% *(n = 3)*	40.0% *(n = 2)*	
Anti-EBV IgG antibodies *(n = 11) ^(1)^*	Present		77.8% *(n = 7)*	22.2% *(n = 2)*	NS (1.000)	11.1% *(n = 1)*	88.9% *(n = 8)*	NS (0.055)
	Absent		100.0% *(n = 2)*	0.0% *(n = 0)*		100.0% *(n = 2)*	0.0% *(n = 0)*	
Anti-CMV IgG antibodies *(n = 11) ^(1)^*	Present		60.0% *(n = 3)*	40.0% *(n = 2)*	NS (0.545)	20.0% *(n = 1)*	80.0% *(n = 4)*	NS (0.545)
	Absent		83.3% *(n = 5)*	16.7% *(n = 1)*		50.0% *(n = 3)*	50.0% *(n = 3)*	
Number of lesions in T2-weighted images on MRI at diagnosis *(n = 26/25) ^(1)^*	0–4		100.0% *(n = 3)*	0.0% *(n = 0)*	NS (0.225)	66.7% *(n = 2)*	33.3% *(n = 1)*	NS (0.696)
	5–9		100.0% *(n = 6)*	0.0% *(n = 0)*		33.3% *(n = 2)*	66.7% *(n = 4)*	
	≥ 10		64.7% *(n = 11)*	35.3% *(n = 6)*		37.5% *(n = 6)*	62.5% *(n = 10)*	
Presence and number of gadolinium-enhanced lesions on MRI at diagnosis *(n = 26/25) ^(1)^*	No		62.5% *(n = 5)*	37.5% *(n = 3)*	NS (0.240)	50.0% *(n = 4)*	50.0% *(n = 4)*	NS (0.632)
	Yes	1–2	90.0% *(n = 9)*	10.0% *(n = 1)*		30.0% *(n = 3)*	70.0% *(n = 7)*	
		3–4	100.0% *(n = 4)*	0.0% *(n = 0)*		25.0% *(n = 1)*	75.0% *(n = 3)*	
		≥ 5	50.0% *(n = 2)*	50.0% *(n = 2)*		66.7% *(n = 2)*	33.3% *(n = 1)*	
Tumefactive lesions on MRI at diagnosis *(n = 26/25) ^(1)^*	Present		76.0% *(n = 19)*	24.0% *(n = 6)*	NS (1.000)	100.0% *(n = 1)*	0.0% *(n = 0)*	NS (0.400)
	Absent		100.0% *(n = 1)*	0.0% *(n = 0)*		37.5% *(n = 9)*	62.5% *(n = 15)*	

(1) Reduced n value due to missing data. (2) Continuous variables are shown as mean + standard deviation (BMI at diagnosis) or median (remainder). Categorical variables are shown as relative and absolute frequency. BMI, body mass index; CMV, Cytomegalovirus; CSF, cerebrospinal fluid; EBV, Epstein–Barr virus; EDSS, Expanded Disability Status Scale; MRI, magnetic resonance imaging; NS, not significant.Bold values are statistically significant.

In the groups defined by the “absence of MRI activity of disease,” we found a statistically significant difference between groups regarding the disease-modifying therapy started at diagnosis (*X*^2^ = 14.899; *p* = 0.006). The association between immunomodulatory treatment and absence of MRI activity of disease was strong (φ_C_ = 0.799). After performing a *post-hoc* analysis, we observed that treatment with natalizumab was positively associated with absence of MRI activity of disease, but treatment with glatiramer acetate had an opposite relationship with the same variable. For both criteria, we did not find any statistically significant differences between groups in the following variables: age at diagnosis, gender, family history of MS, BMI at diagnosis, number and topography of clinical relapses occurred previously to diagnosis, EDSS score at diagnosis, existence of chronic diseases, oligoclonal bands in CSF, anti-EBV and anti-CMV IgG antibodies, number of lesions in T2-weighted images, gadolinium-enhanced lesions or tumefactive lesions on MRI (at diagnosis).

## Discussion

POMS is, as previously mentioned, a condition characterized by the existence of a high inflammatory activity within the CNS, which is clearly reflected in the high lesion burden that children and adolescents normally present at any stage of their disease (Renoux et al., 2007; Brenton et al., 2020; Fisher et al., 2020; De Meo et al., 2021). This aspect is not innocent, regarding MS natural history, as high clinical and radiological activity, in its earlier stages, is related to a more unfavorable prognosis in the medium and long term (Renoux et al., 2007; Yeshokumar et al., 2017; Barraza et al., 2021). In this way, the identification of clinical or paraclinical elements that may function as predictors of short-term disease control can constitute a useful tool, in practical terms, even for managing the type of therapeutic intervention to be implemented. As already mentioned, NEDA-3 is an interesting metric in defining this treat-to-target strategy and there is already literature that corroborates its practical applicability (Diem et al., 2018; Prosperini et al., 2018; Freedman et al., 2019; Margoni et al., 2020). In our study, NEDA-3 was achieved by 1/3 of the participants included, at 12 months after MS diagnosis. It is important to emphasize that cases identified between 2010 and 2021 were recruited for the analysis, which, given this time span, immediately introduced some bias to the type of drugs used in the treatment of these children and adolescents. The PARADIGMS trial, which allowed the approval of fingolimod for the treatment of POMS, from 10 to 17 years old, was only published in 2018 (Chitnis et al., 2018) and before that, the prescription of drugs considered first-line immunomodulators (less effective, but safer), was classically considered (Ghezzi et al., 2010). But even though the profile of treatments used may be heterogeneous among individuals, the characteristics of the population analyzed in our study are consistent with what is most frequently described in the literature for POMS. Most participants were females and had a mean age at diagnosis of 14.8 years, which is in line with the known epidemiology for the disease, particularly after puberty (Yeshokumar et al., 2017). All participants presented a relapsing–remitting form of MS, fact that corroborates the predominance of this phenotype in POMS (Renoux et al., 2007; Fisher et al., 2020). The median EDSS score at diagnosis was relatively low (1.5 out of 10) and 65.4% of the patients presented 10 or more lesions in T2-weighted images on the baseline MRI at diagnosis. Both facts support the evidence of a meaningful lesion burden in POMS, although not consummated in clinical disability (clinical-radiological dissociation). These results reinforce the prominent role of inflammation in the pathophysiology of this condition (Brenton et al., 2020), but it is interesting that we actively looked for the presence of tumefactive lesions (which reinforce the aggressiveness of inflammation inside the CNS) in these patients and found only 1 case in our cohort (Barraza et al., 2021). Nevertheless, in the group of patients that did not fulfill conditions for NEDA-3, most (88.9%) did not achieve the “absence of MRI activity of disease” criterion, although all of them did not show progression in neurological disability. This data exemplifies once again the influence of inflammation in POMS mechanisms, as well as neuroplasticity verified in pediatric age (Fisher et al., 2020). We think that this information is also very relevant to understand the apparent failure related to the use of glatiramer acetate in controlling the radiological activity of the disease. This is a very safe drug, but less effective (theoretically) than other treatment options available today, with a greater impact on controlling inflammation. At a time when no drug was formally approved for the treatment of children and adolescents, it is understandable to resort to drugs that, above all, were safe and did not cause significant adverse effects. However, given the known inflammatory nature of the disease, it was expected that its ability to stop the accumulation of lesions on the MRI would not be so significant, regardless of the occurrence of relapses. As the years went by, we witnessed a progressive change in the medical team’s prescribing habits, which favored the use of much more effective drugs, albeit with a more sensitive profile of adverse effects.

After analyzing these results, it is plausible to consider new lesions on follow-up MRI as red flags that can point out the need for serial imaging studies and more aggressive therapeutical approaches, to obtain a more assertive control of disease evolution. By comparing our variables’ distribution between groups defined by NEDA-3 status at 12 months after diagnosis, the results showed that patients under treatment with natalizumab (monoclonal antibody that is a selective inhibitor of adhesion molecules, binding to integrins’ α4 subunit), usually considered a second line agent, with no formal and published clinical trials that corroborate its efficacy and safety in pediatric patients (Palavra et al., 2021), had a higher probability of achieving NEDA-3 (*p* = 0.017). It is still interesting to reflect on this therapeutic choice: despite the absence of a formal indication, 4 patients out of 27 were treated with natalizumab, since they had a disease defined as being aggressive, clinically, or imagiologically. And, in such an inflammatory condition, this choice proved to be useful, as it turned out a predictor of reaching a NEDA-3 state after 12 months of treatment. These results are in accordance with the ones obtained in the national study TyPed (Palavra et al., 2021), which corroborated the efficacy and safety of natalizumab in POMS, and in a study by Margoni et al. (2020), that also verified the efficacy of this drug in the same clinical condition (although considering a NEDA-4 status, which also accounts for absence of cognitive deficits and brain atrophy on MRI).

Restringing the analysis to each one of the three NEDA-3 criteria, we did not find any statistically significant differences in the “absence of clinical relapses” criterion, and we did not study the “absence of progression in neurological disability” criterion, since it was achieved by the entire cohort. Actually, scientific evidence points out that 90% of children with MS do not show physical disability during the first 5 years after the initial demyelinating event (Yeshokumar et al., 2017). In the groups defined by the “absence of MRI activity of disease” criterion, we observed that treatment with natalizumab was positively associated with NEDA-3 achievement. On the other hand, treatment with glatiramer acetate (a classical first line drug) was associated with a higher chance of not achieving that metric. Although surprising, this finding is easily explained due to a selection bias for treatment, especially in younger patients (age group below 10 years old, in which there are no formally approved drugs), and with lower lesion burden (therefore with a less aggressive disease, being plausible to start with a safer drug with fewer adverse side effects). The use of a potent anti-inflammatory drug, such as natalizumab, in an early stage of the disease, thus seems to predict a better clinical response, translated into a greater probability of reaching the NEDA state.

Considering the relevance that EBV has assumed in the pathophysiology of MS (Soldan and Lieberman, 2023), it was our purpose to also include the analysis of variables related to serology for this virus, trying to understand whether contact with it could motivate a different probability (or not) of reaching NEDA-3 status 1 year after diagnosis. It is known that primary EBV infection usually occurs in children and that the immune response against it is usually robust. However, EBV promotes a chronic and latent infection of B lymphocytes, which deactivates many of the innate and adaptive control mechanisms, with a natural impact on the risk of developing immune-mediated conditions (Soldan and Lieberman, 2023). As examples of this, it can be mentioned that Epstein–Barr virus nuclear antigen 1 (EBNA1) induces the recruitment of regulatory T cells through CXCL12 secretion, and also a suppression of the activity of NK cells; EBNA2 activates the transcription of pro-inflammatory genes (such as those encoding TNF, PDL1, IL-18R and alfa-lymphotoxin) and suppresses interferon responses; and EBV miRNAs suppress the activity of CD8^+^ T cells (Soldan and Lieberman, 2023). In this way, the prevention of EBV infection could become a therapeutic target and research has been conducted to assess the impact of vaccines directed against this infection (using, for example, antibodies against components of the viral entry proteins) at risk of developing MS or another type of immune-mediated disease, including some forms of lymphoma (Soldan and Lieberman, 2023).

All the individuals of our study that showed negativity for anti-EBV IgG antibodies (i.e., without previous infection by this viral agent) achieved NEDA-3 at 12 months after MS diagnosis, fact sustained by a statistically significant association between both variables (*p* = 0.018). Current scientific evidence supports the hypothesis that a previous EBV infection is a risk factor for developing MS (Soldan and Lieberman, 2023) and that higher antibody titers predispose to a more significant MRI activity of disease (Farrell et al., 2009) and a lower NEDA-3 achievement (Dominguez-Mozo et al., 2020). However, to our knowledge, an association between the absence of anti-EBV IgG antibodies and NEDA-3 achievement in POMS has not been previously reported. Regardless of not being statistically significant, the differences observed on the variables “existence of chronic diseases” (between groups defined by NEDA-3) and “anti-EBV IgG antibodies” (between groups defined by the “absence of MRI activity of disease” criterion) assumed *p* values at the limit of significance (*p* = 0.059 and *p* = 0.055, respectively). Therefore, further studies in broader samples may clarify the eventual predictive nature of these variables.

Our study has some limitations worth considering: it has an unicentric and retrospective design, which allowed us to include a sample of reduced dimensions, with high susceptibility to missing data, diminishing the statistical power of our analysis and introducing a potential selection bias; the period defined for evaluating cases (12 months after diagnosis) is short and, naturally, a longer follow-up will be necessary to reach stronger conclusions, particularly with regard to the longer-term effectiveness of natalizumab; additionally, evidence (Yokote et al., 2018) has been indicating that NEDA-3 status may not be sufficient as a prognostic tool of evolution to long-term neurological disability–the measure of brain atrophy has been recognized as a more precise biomarker, included in the concept of NEDA-4 status (Beadnall et al., 2019). The conduction of further research in this field, particularly with a prospective and multicentric design, assumes great value to potentiate the statistical power of the associations that we observed. It would also be relevant to analyze if these associations persist when evaluating NEDA-3 achievement in a long-term basis, difficulty which is reported by some research made with adult populations (Rotstein et al., 2015). In the future, the use of NEDA-4 status or of new biomarkers (Disanto et al., 2017; Beadnall et al., 2019), like serum neurofilament light chains (sNfL), may be seen as additional tools to be integrated into the clinical equation of the follow-up of children and adolescents with MS.

## Conclusion

In this single-center, retrospective and exploratory study, with a small number of participants, we observed that the negativity for anti-EBV IgG antibodies and treatment with natalizumab were associated with a higher probability of achieving NEDA-3 status, at 12 months after POMS diagnosis. The benefit of treatment with the monoclonal antibody persisted even when we only studied the “absence of MRI activity of disease” criterion, in an analysis of each of the aspects that define NEDA-3 status. Our results suggest, with the limitations previously mentioned, that an early and more aggressive therapeutical approach may be important to control disease evolution, taking advantage of pediatric neuroplasticity. The application of these predictors as risk-stratification and personalized therapy tools may be considered in the future, aiming at an optimization of the approach to POMS and to patients’ quality of life. Further multicentric and prospective studies, in larger samples, are essential to confirm the strength of our results. It is our objective to replicate the protocol in a more comprehensive study (also including environmental, geographic and lifestyle aspects in the analysis), involving other national and international centers, so that, by increasing the number of participants, we can obtain more robust and solid results.

## Data availability statement

The original contributions presented in the study are included in the article/supplementary material, further inquiries can be directed to the corresponding author.

## Ethics statement

The studies involving humans were approved by Local Ethics Committee–Centro Hospitalar e Universitário de Coimbra. The studies were conducted in accordance with the local legislation and institutional requirements. Written informed consent for participation in this study was provided by the participants’ legal guardians/next of kin.

## Author contributions

FP: Conceptualization, Data curation, Investigation, Methodology, Project administration, Resources, Software, Supervision, Validation, Writing – original draft, Writing – review and editing. DS: Formal analysis, Investigation, Software, Writing – original draft. CF: Data curation, Investigation, Writing – original draft. RF: Conceptualization, Investigation, Methodology, Software, Writing – review and editing. MV: Data curation, Writing – review and editing. CP: Writing – review and editing. CC: Data curation, Writing – review and editing. JR: Data curation, Writing – review and editing. JA: Data curation, Writing – review and editing. CR: Data curation, Supervision, Writing – review and editing.

## Funding

The authors declare that no financial support was received for the research, authorship, and/or publication of this article.

## Conflict of interest

The authors declare that the research was conducted in the absence of any commercial or financial relationships that could be construed as a potential conflict of interest.

## Publisher’s note

All claims expressed in this article are solely those of the authors and do not necessarily represent those of their affiliated organizations, or those of the publisher, the editors and the reviewers. Any product that may be evaluated in this article, or claim that may be made by its manufacturer, is not guaranteed or endorsed by the publisher.
